# Prevalence of class 1, 2 and 3 integrons among multidrug-resistant *Pseudomonas aeruginosa* in Yazd, Iran

**Published:** 2018-10

**Authors:** Mohadeseh Zarei-Yazdeli, Gilda Eslami, Hengameh Zandi, Masoumeh Kiani, Kazem Barzegar, Hanieh Alipanah, Seyed Morteza Mousavi, Marzieh Shukohifar

**Affiliations:** 1 Department of Microbiology, Faculty of Medicine, Shahid Sadoughi University of Medical Sciences, Yazd, Iran; 2 Department of Parasitology and Mycology, Faculty of Medicine, Shahid Sadoughi University of Medical Sciences, Yazd, Iran; 3 Research Center for Food Hygiene and Safety, Shahid Sadoughi University of Medical Sciences, Yazd, Iran; 4 Department of English Language, School of Medicine, Shahid Sadoughi University of Medical Sciences, Yazd, Iran; 5 Department of Biology, Borujerd Branch, Islamic Azad University, Borujerd, Iran; 6 Department of Biostatistics and Epidemiology, Faculty of Paramedicine Abarkouh, Genetic and Environmental Adventures Research Center, Shahid Sadoughi University of Medical Sciences, Yazd, Iran

**Keywords:** *Pseudomonas aeruginosa*, Antimicrobial resistance, Class 1 integron, Class 2 integron, Class 3 integron

## Abstract

**Background and Objectives:**

Antibiotic resistance in *Pseudomonas aeruginosa* is an increasing health problem. Integrons are associated with a variety of gene cassettes, which confer resistance to multiple classes of antibiotics. This study aimed at screening the presence of class 1, 2 and 3 integrons in *P. aeruginosa* in Yazd, Iran.

**Materials and Methods:**

This study was carried out on *P. aeruginosa* strains from March 2016 to March 2017. Clinical specimens were initially identified by the standard biochemical methods and their resistance patterns to antibiotics were studied using the disc diffusion method. PCR was carried out for the detection of class 1, 2 and 3 integrons using *intI1*, *intI2* and *intI3* gene primers, respectively.

**Results:**

Antimicrobial susceptibility test showed that 75% of isolates were detected as multi-drug resistant (MDR), and lowest resistance was observed in ciprofloxacin (48.6%) and most resistance was in gentamicin (63.2%). Moreover, PCR results showed that 22 (15.3%) and 119 (82.6%) of *P. aeruginosa* isolates carried *intI2* and *intI1* genes, but *intI3* gene was not found.

**Conclusion:**

Since it is customary to observe Class I integrons in *P. aeruginosa* isolated from clinical samples, they are often responsible for antibiotic resistance gene transfer, which calls for evaluation of integrons as contributing factors in antibiotic resistance.

## INTRODUCTION

*Pseudomonas aeruginosa* are the leading Gram-negative pathogen bacteria associated with nosocomial infections (1). *P. aeruginosa* is responsible for 10–15% of the nosocomial infections worldwide (2). Antimicrobial resistance is a worldwide emerging problem (3), and the widespread use of antibiotics is probably the main reason for the increase in multidrug resistance (MDR) among *P. aeruginosa* strains (4). Besides, in many bacteria, exchangeable genetic elements such as plasmids, transposons and integrons are responsible for the dissemination of antibiotic resistance (5). Integrons are common systems of gene capture and expression that incorporate open reading frame and convert them into functional genes. Integrons are mainly composed of the promoter, attachment site (*att I*) and integrase gene (*int I*) (6). There has long been an association between three antibiotic resistant integron classes (including classes 1 to 3) and MDR phenotypes (7–8), where classes are determined based on sequence differences in the respective *intI* gene (9). However, class 1 integrons have been found to be the most prevalent in clinical isolates, carrying single or multiple gene cassettes, which confer resistance to aminoglycosides, β-lactams, chloramphenicol, and macrolides (10–11). Class 2 integrons are not as widespread among bacteria, even though class 2 integrons are associated with a mobile DNA element, the Tn7 transposon (12). Arakawa recognized a class 3 integron-mediating *IMP-1* for the first time in an *S. marcescens* strain isolated in Japan in 1995, which is one of the few instances of carriage of that class of integrons reported so far (13). Class 2 and class 3 integrons contain the integrase genes (*intI2* and *intI3*), whose products are 46% and 61% identical to class 1 integrase, respectively (5). In this respect, prevalence of integrons is variable in different parts of the world. For example, studies carried out in Malaysia, China, and Iran (14–16) showed respectively that 63%, 38% and 35.6% of isolated *P. aeruginosa* carry class 1 integron gene.

The aim of the present study was to find out the molecular relation and the existence of integrons with multidrug resistance pattern of *P. aeruginosa* strains isolated from clinical specimens in Yazd, Iran.

## MATERIALS AND METHODS

### Specimen collection and identification.

This descriptive study was carried out on 144 clinical isolates of *P. aeruginosa* which were collected from Shahid Sadoughi Hospital, Yazd, from March 2016 to March 2017. A questionnaire was used for recording patient demographic data such as: name, age, sex, type of sample, and ward. Bacterial isolates were recovered from different clinical specimens such as blood, bronchial fluid, urine, cerebrospinal fluid, catheter, pleural fluid, ear swap, sputum, and wound and inoculated on sheep blood agar and EMB agar media before incubation at 37°C for 24 h. All isolates were identified as *P. aeruginosa* using standard biochemical tests like growth on Cetrimide agar medium (Liofilchem, Italy), growth at 42°C, oxidase test, gram stain, pigment production, and Oxidation/Fermentation test.

### Antimicrobial susceptibility testing.

Antimicrobial susceptibility testing were performed using the disk diffusion method according to CLSI guidelines (17) for gentamicin (10 μg), amikacin (30 μg), tobramycin (10 μg), imipenem (10 μg), piperacillin (100 μg), ticarcillin (75 μg), ceftazidime (30 μg), and ciprofloxacin (5 μg). All antibiotics were obtained from Mast Company, England. *P. aeruginosa* ATCC 27853 was used as a control.

### PCR assay.

Extraction of genomic DNA from *P. aeruginosa* isolates was performed by salting out method and were stored at −20°C until use (18). Specific primers were developed for each gene using Primer 3 (Table 1). The final optimized PCR reaction consisted of 0.5 μl MgCl_2_ (100 mM), 0.5 μl dNTP (10 mM), 0.2 μl (1 unit) Taq DNA polymerase (Cinnagen, Iran), 1 μl of each primer (10 pmol) (Alpha DNA, Canada), 2.5 μl PCR buffer (10 X), and 0.5 μl of DNA template (100 μg/ml) in total volume of 25 μl with double distilled water. DNA amplification was performed in the thermo cycler (Quanta Biotech, England) using an initial denaturation step for 5 min at 94°C (one cycle), followed by 35 cycles of 1 min at 94°C, 1 min at 50°C and 1min at 72°C for *intI1*. The above condition was used for *intI2* and *intI3* the same as the one for *intI1* but in annealing temperature at 47°C and 52°C, respectively. All of the reactions were finalized for 5 min at 72°C (one cycle). Amplicons were analyzed by electrophoresis on 1% agarose gels at 5V/Cm alongside with 50 bp DNA ladder. To confirm the amplicons, some isolates were sequenced and analyzed with bioinformatics software such as BLAST and ClustalW2 (20).

**Table 1.** Primers used for amplification of class 1, class 2 and class 3 integrase genes in *P. aeruginosa* clinical isolates.

**Primer**	**Primer sequence (5′-3′)**	**Product size (bp)**
*intI1*-F	5′- GGTGTGGCGGGCTTCGTG-3′	480
*intI1*-R	5′- GCATCCTCGGTTTTCTGG-3′	480
*intI2*-F	5′- CTAGAATAGGCTGTATAGGCAGA-3′	850
*intI2*-R	5′- GAGTGACGAAATGTATGACAAG-3′	850
*intI3*-F	5′- CAGTCTTTCCTCAAACAAGTG-3′	702
*intI3*-R	5′- TACATCCTACAGACCGAGAAA-3′	702

### Statistical analysis.

The data was analyzed using the SPSS18. The Chi-square test was employed to calculate the P-value in terms of resistant numbers of integron-positive and integron-negative isolates (*P*<0.05).

## RESULTS

Seventy-nine out of 144 patients (54.9%) were male and 65 (45.1%) were female. Their age ranged from one month to 79 years with a mean of 34.9+22.7 (+SD) years. The isolates were obtained from different clinical specimens including burn wounds (43.8%), urine (23.6%), tracheal fluid (13.9%), wound (6.9%), blood (6.3%), sputum (2.8%), and catheter (2.8%). The isolates were obtained from hospitalized patients in burn ward (47.2%), ICU units (18.8%), internal (16.7%), general surgery (12.5%), and neurology (4.9%) wards. *P. aeruginosa* isolates showed the most resistance against gentamicin (63.2%) and imipenem (62.5%), respectively. The lowest resistance rates were seen against ciprofloxacin (48.6%) and piperacillin (54.9 %), respectively. One hundred and eight strains (75%) were resistant to more than three groups of antibiotics (MDR). Forty strains (27.7%) were resistant to all tested antibiotic groups.

**Fig. 1.**
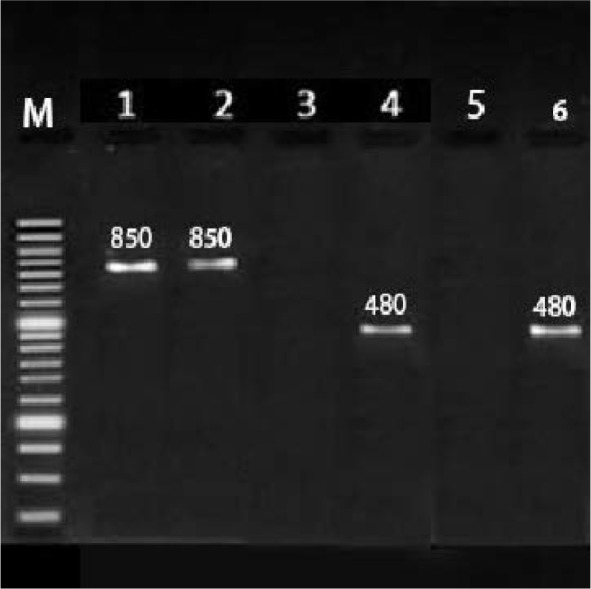
PCR amplification of Class 1, 2 and 3 integron genes in clinical *P. aeruginosa* isolates. Lane M, 50 bp DNA ladder; lanes 1, positive controls for *intI2* (850 bp); lane 2, isolates positive for *intI2* (850 bp); lane 3, negative controls for *intI2*; lanes 4, positive controls for *intI1* (480 bp); lane 5, negative control for *intI1*; lane 6, isolates positive for *intI1* (480 bp).

Class 1 integron was detected in 119 (82.6%) isolates (Fig. 1), of which 59.7% were multidrug-resistant and 22.9% were intermediate or sensitive to tested antibiotics. Class 2 integron was found in 22 (15.3%) *P. aeruginosa* isolates (Fig. 1) of which 14.58% were multidrug-resistant, 0.7% were intermediate or sensitive, and 19 (13.19%) strains had both *intI1* and *intI2* genes. No *intI3* gene was detected in any of the isolates. A significant correlation was observed between the presence of integrons class 1 and resistance against gentamicin (Table 2), and also integrons class 2 and resistance against ceftazidime, amikacin and tobramycin (Table 3). Moreover, there was a significant correlation between burn unit and the presence of class 1 integrons.

**Table 2.** Comparison of the antibiotic resistance between class 1 integron -positive and class 1 integron -negative of *P. aeruginosa*

**Antibiotics**	**Antibiotic resistance No. (%)**	**Integron 1-negative No. (%)**	**Integron 1-positive No. (%)**	**P value^a^**	**OR**
Amikacin	84 (58.3%)	16 (19%)	68 (81)	0.14	0.538
Ceftazidime	82 (56.9)	16 (19.5)	66 (80.5)	0.373	0.776
Tobramycin	80 (55.6)	12 (15)	68 (85)	0.268	1.444
Imipenm	90 (62.5)	21 (23.3)	69 (76.7)	0.07	0.362
Ticarcillin	80 (55.6)	15 (18.8)	65 (81.3)	0.678	0.830
Gentamicin	91 (63.2)	21 (23.1)	70 (79.9)	0.012	0.272
Ciprofloxacin	70 (48.6)	12 (17.1)	58 (82.9)	0.14	0.548
Piperacillin	79 (54.9)	14 (17.7)	65 (82.3)	0.544	1.048

**Table 3.** Comparison of antibiotic resistance between class 2 integron -positive and class 2 integron -negative of *P. aeruginosa*

**Antibiotics**	**Antibiotic susceptibility R, No. (%)**	**Integron 2-negative isolates R, No. (%)**	**Integron 2-positive isolates R, No. (%)**	**P value^a^**	**OR**
Amikacin	84 (58.3%)	66 (78.6%)	18 (21.4)	0.022	0.43
Ceftazidime	82 (56.9)	64 (78.0)	18 (22.0)	0.03	0.48
Tobramycin	80 (55.6)	63 (78.8)	17 (21.2)	0.021	3.18
Imipenm	90 (62.5)	71 (78.9)	19 (21.1)	0.4	0.9
Ticarcillin	80 (55.6)	66 (82.5)	14 (17.5)	0.6	0.24
Gentamicin	91 (63.2)	76 (83.5)	15 (16.5)	0.3	1.2
Ciprofloxacin	70 (48.6)	55 (78.6)	15 (21.4)	0.08	0.76
Piperacillin	79 (54.9)	63 (79.7)	16 (20.3)	0.16	0.58

### Nucleotide sequence accession numbers.

The nucleotide sequence data reported in this work was assigned to the NCBI/GenBank nucleotide sequence database under accession no. KF146819 and KF358999.

## DISCUSSION

Currently, in the investigation of genetic bases of *P. aeruginosa* multi-resistance, an important aspect that has been considered is the integron and the associated gene cassettes (19). The role of these elements in the horizontal acquisition and expression of genes, and as a gene reservoir, has been associated with the emergence of antibiotic resistance among clinical isolates of bacteria (21). The presence of populations of MDR strains among clinical isolates is a cause of concern for physicians applying empirical treatment, especially in serious cases of *P. aeruginosa* infections.

The results of our study showed that 40 strains (27.7%) of *P. aeruginosa* isolated from our hospital were resistant to all commonly used antibiotics. Based on studies by Kohanteb (22) and Nikokar (23), it was found that 26.7% and 19.7% of the *P. aeruginosa* isolates were resistant to all anti-*pseudomonas* antibiotics. Our results are consistent with the findings of other studies. Furthermore, the results of this study showed that there were MDR *P. aeruginosa* strains disseminated through different clinical wards in our hospital, indicating lack of appropriate supervision on this issue at this hospital, thus the infection control measures should be applied to prevent the transmission of *P. aeruginosa* strains.

Different definitions have been employed to characterize multidrug resistant (MDR) isolates of *P. aeruginosa* in biomedical publications (24). In the majority of studies, MDR was defined as the acquired non-susceptibility to at least one agent in three or more antimicrobial categories, mainly aminoglycosides, anti-pseudomonal penicillins, cephalosporins, carbapenems, and fluoroquinolones (25–26). Our study revealed that the frequency of MDR *P. aeruginosa* is 75%. Some studies carried out in Amazon region in Brazil (21), Zhenjiang in China (27) and Guilan in (23) showed that 75%, 90.1% and 42.3% of *P. aeruginosa* isolates were resistant to three or more antimicrobials, respectively.

Considering that almost half of the samples were taken from a burn unit and 18.8% of the samples were collected from an ICU unit, the high prevalence of MDR cases can be justified in the present study. In this study, the highest antimicrobial resistance rate was observed for gentamicin (63.2%). The resistance rate against gentamicin was reported 55.8% in France in 2008 (28) and 95% in Thailand in 2013 (29) and in Iran, in studies carried out by Kohanteb and Imani, these rates were 68.3% and 25.5%, respectively (22, 30).

On the whole, resistance to aminoglycosides in developing countries was more than that in developed countries which could be the result of the indiscriminate use of antibiotics. In our study, class 1 integron was detected in 82.6% of the isolates. Reports of *P. aeruginosa* clinical isolates carrying class 1 integron in Iran vary between 39.4% and 56.3% (31, 23, 32). Other studies carried out on various clinical samples in Amazon region in Brazil (21), Malaysia (14), Nanjing (15) and Zhenjiang (27) in China showed that 41.5%, 63%, 38% and 40.8% of *P. aeruginosa* isolates carried the class 1 integron gene, respectively. Comparing the results of our study with those from other studies, the increased prevalence of integrase 1 is obvious which can be the result of geographical differences and indiscriminate use of antibiotics.

In the present study, the class 1 integron in MDR strains has higher prevalence as compared with the non-MDR strains; these results are consistent with those of Yousefi and Zhenjiang study (27, 31). Our study also revealed that class 1 integron was significantly associated with resistance to given antibiotics, including aminoglycosides, quinolones, and β-lactam compounds. In view of the fact that many antibiotic resistance gene cassettes encoding resistance to a wide range of antibiotics in *P. aeruginosa* are carried by class 1 integron, this is not a wonder. Resistance against the antibiotics was also observed in other integron-negative isolates, however. Chromosomal-encoded enzymes or other mobile elements could account for the acquisition of the antibiotic-resistance genes of the isolates.

In this study, the *intI2* gene was detected in 15.3% of the isolates. The research done by Xu (33) in China and Moazami Goudarzi (20) in Iran estimated the prevalence of class 2 integron to be 19.5% and 2.7%. In the research conducted in Thailand (34), *intI2* and *intI3* genes were not present. Furthermore, the *intl3* gene was not observed in this research. On the other hand, identification of *intI3* has been reported before in three or more Gram-negative bacteria species that had been isolated from areas with significant geographical difference. Therefore, the emergence and proliferation of integrons of class 3 carry a variety of gene cassettes that explain multiple antimicrobial resistance can be regarded as a global issue rather than a local problem (35).

## CONCLUSION

In this study, we observed high prevalence of MDR *P. aeruginosa* (75%). In order to prevent formation of *P. aeruginosa* strains which may be MDR, an antimicrobial susceptibility test, especially MIC, should be performed before starting the treatment, and adequate supervision is required for the use of antibiotics. Regarding the high prevalence of *intI1* gene in this study (82.6%) and its effect on increasing the *P. aeruginosa* antibiotics resistance, determination of positive cases and precise detection of antibiotic susceptibility pattern is strongly recommended. Apart from whether the resistance genes are present in integrons, in this study there is noticeable relationship between the presence of integrons and increased resistance to many groups of antibiotics and this could be a concern because these structures can alter involved genes in resistance between strains so that these strains become resistant to new antibiotics.
